# Supplementary material for: I won’t listen if I think we’re losing our way: How right-wing authoritarianism affects the response to different anti-prejudice messages
Source: PLoS One. 2023 Jan 17;18(1):e0280557. doi: 10.1371/journal.pone.0280557 (PMC9844905; doi:10.1371/journal.pone.0280557)
Supplement: Attachment — Submitted filename: Revision letter.1.docx [file pone.0280557.s001.docx]

Dear Editor,

Thank you for the opportunity to revise this manuscript. Below, we address each of the reviewer’s comments and editor’s requests.

**Editor’s requests:**

*1. Please ensure that your manuscript meets PLOS ONE's style requirements, including those for file naming. The PLOS ONE style templates can be found at*

[*https://journals.plos.org/plosone/s/file?id=wjVg/PLOSOne_formatting_sample_main_body.pdf*](https://journals.plos.org/plosone/s/file?id=wjVg/PLOSOne_formatting_sample_main_body.pdf)  *and*

[*https://journals.plos.org/plosone/s/file?id=ba62/PLOSOne_formatting_sample_title_authors_affiliations.pdf*](https://journals.plos.org/plosone/s/file?id=ba62/PLOSOne_formatting_sample_title_authors_affiliations.pdf)

Thank you for these suggestions. We have addressed this.

*2. Thank you for stating the following financial disclosure:*

*“The funders had no role in study design, data collection and analysis, decision to publish, or preparation of the manuscript.”*

*At this time, please address the following queries:*

*a)        Please clarify the sources of funding (financial or material support) for your study. List the grants or organizations that supported your study, including funding received from your institution.*

*b)        State what role the funders took in the study. If the funders had no role in your study, please state: “**The funders had no role in study design, data collection and analysis, decision to publish, or preparation of the manuscript.”*

*c)        If any authors received a salary from any of your funders, please state which authors and which funders.*

*d)        If you did not receive any funding for this study, please state: “The authors received no specific funding for this work.”*

*Please include your amended statements within your cover letter; we will change the online submission form on your behalf.*

Thank you for these suggestions. We have amended the cover letter accordingly.

3. Please include your full ethics statement in the ‘Methods’ section of your manuscript file. In your statement, please include the full name of the IRB or ethics committee who approved or waived your study, as well as whether or not you obtained informed written or verbal consent. If consent was waived for your study, please include this information in your statement as well.

We have added this as a section, labelled ethics.

*4. In your Data Availability statement, you have not specified where the minimal data set underlying the results described in your manuscript can be found. PLOS defines a study's minimal data set as the underlying data used to reach the conclusions drawn in the manuscript and any additional data required to replicate the reported study findings in their entirety. All PLOS journals require that the minimal data set be made fully available. For more information about our data policy, please see* [*http://journals.plos.org/plosone/s/data-availability*](http://journals.plos.org/plosone/s/data-availability)*.*

*Upon re-submitting your revised manuscript, please upload your study’s minimal underlying data set as either Supporting Information files or to a stable, public repository and include the relevant URLs, DOIs, or accession numbers within your revised cover letter. For a list of acceptable repositories, please see* *http://journals.plos.org/plosone/s/data-availability#loc-recommended-repositories. Any potentially identifying patient information must be fully anonymized.*

*Important: If there are ethical or legal restrictions to sharing your data publicly, please explain these restrictions in detail. Please see our guidelines for more information on what we consider unacceptable restrictions to publicly sharing data: http://journals.plos.org/plosone/s/data-availability#loc-unacceptable-data-access-restrictions. Note that it is not acceptable for the authors to be the sole named individuals responsible for ensuring data access.*

*We will update your Data Availability statement to reflect the information you provide in your cover letter.*

We have added the URL in the cover letter.

<https://osf.io/wxjcd/>

*Please review your reference list to ensure that it is complete and correct. If you have cited papers that have been retracted, please include the rationale for doing so in the manuscript text, or remove these references and replace them with relevant current references. Any changes to the reference list should be mentioned in the rebuttal letter that accompanies your revised manuscript. If you need to cite a retracted article, indicate the article’s retracted status in the References list and also include a citation and full reference for the retraction notice.*

We have checked and found all studies cited.

Reviewer 1:

*Reviewer #1: The authors address an important topic: the degree to which individuals who are high in right wing authoritarianism (RWA) are more or less sensitive to prejudice reduction campaigns. This speaks to a growing body of literature seeking to understand what kinds of prejudice-reduction interventions may be most effective in diverse contexts. That said, below I raise several concerns the authors should address before publication:

1) The authors sample size seems quite small, with no group containing more than 100 subjects. To what degree are the authors null findings a true null or just a consequence of the study being underpowered?*

We did a power analysis prior to running the study, but due to word count, we removed this. We have restored this in the manuscript, lines 217-222.

To provide a little more detail than in the manuscript, we calculated the effect size by looking at the relevant t-test in the manuscript (Dru, 2007):
“The regression coefficient for RWA in the group values condition was marginally divergent from the one calculated for the competitive condition (t(118)= 1.89, p=.06). “ That converts to a cohen’s D of .35, or effect size of F= 0.175.

We now say in the revised manuscript:

In November 2018, Americans who voted in the 2016 Election from Amazon’s Mechanical Turk (N=388 (retained), Mage= 36.11 years old, 60.1% Male) were invited to participate in a short, online survey titled “Understanding social cohesion and intergroup attitudes”. We had aimed to recruit at least 396 participants, based our sample size calculation on Dru’s 2007 study (12), which ran an anti-prejudice experiment while examining RWA. They found an effect size of F= 0.175 (df=188), for a similar intervention. Our power analysis for an ANOVA using the same effect size (a=.05, power=.80) suggested we needed 360 participants, but we aimed to over recruit by 10% in order to remove those who failed attention checks.

*2) Recent research suggests that as of 2018, as many as 25% of respondents participating on MTurk were found to be fraudulent. What checks have the authors done to ensure that their respondents are genuinely participating?*

We had multiple attention checks and manipulation checks. Here, we have clarified the content of one of the attention checks.

We now revised the manuscript as follows:

Manipulation and attention check

We had three manipulation/attention check questions. These manipulation checks asked participants about their comprehension about the messages, asking who the message source was, their views, and who the interviewer was. If anyone failed the attention check, they were presented with the messages again, with a timer that would not let them proceed until one minute had passed. We had 16 participants who failed at least two attention checks (not included in the N=388). Half of these participants also showed a lack of consistency in their responses in scales, with most completing the scales in a near impossible timeframe (i.e., in a matter of seconds). One quarter of these participants also failed a simple attention check where they were told to select a specific response to the question (e.g., “please select the option that shows you do not accept a given statement”). One of these participants stated that they disliked the survey and deliberately manipulated their responses to make it unlikely for us to find anything. These participants were removed from the analyses. We also asked participants about their knowledge of the Iraq war. 100% of participants were aware of the Iraq war.

*3) How much variation is there in RWA in the sample? Does splitting the sample at the median appropriately capture differences between high and low RWA participants?*

There was slightly more variation in the sample at the high level of RWA, while low levels of RWA had considerably less variance (i.e., it was positively skewed). The median was 4, with the lowest possible score of 1 and the highest possible score of 8. This suggests that those low in RWA were fairly similar, while those high in RWA were had a bigger range. We have provided a bit more detail in the manuscript about this (ln 282-286)

A median split for RWA (Median=4.00) was conducted to enable comparison between high RWA and low RWA groups; this fit the data as there was clustering at the bottom half of the scale (suggesting similarity), with more extremity at the top end. This was reflected in higher variance in the top half of scores for RWA (SD= .934) than in the lower half (SD=.622)

Reviewer 2:

*The authors investigate an important topic and provide sufficient justification based on prior literature. There is only partial support for their various hypotheses which limit the contributions of this paper. I would suggest the authors address thee things in a revision:
1) The authors assume in-group membership for the in-group stimulus member but have no manipulation check to ensure he was perceived as an in-group member. This is an important limitation and should be explicit.*

*2) Related to number one above, it is overly simplistic to assume one chosen in-group and one chosen out-group member will be representative of either. Egon Brunswick argued that we should use a sample of stimulus for the same reasons we use samples of participants. It is a huge assumption to assume that the single in-group member is typical or representative of participant in-group members.*

We have added a paragraph to make these limitations clearer (ln 450-464):

Extending on this same limitation, there is a possibility that the ingroup member was not seen as “ingroup enough”. We did not have a manipulation check to assess whether or not participants saw ingroup member as being relatively similar. We assumed that the ingroup member would be influential due to shared identity, but this was not assessed. We also assumed that the mere presence of the outgroup member would shift that ingroup member to become more representative, but this was an assumption based on theoretical and previous evidence, rather than being tested in this study. This limitation could be addressed in future research in two ways. First, future research should pilot multiple ingroup prototypes that cover a range of ingroup norms, and attempt to align specific prototypes against the norms espoused by participants. For example, a rural American may respond better to a prejudice intervention by someone espousing values more common in rural areas. Second, future research should assess how “ingroup” a prototype is, before presenting a message from that member. Together, these may lead to more powerful interventions that are more custom fit, thereby potentially improving their impacts on those traditionally resistant to anti-prejudice messages.

*3) The authors should review more literature on "anti-Muslim attitudes" and "authoritarianism." There are significant works not addressed by the authors.*

We have now discussed more literature on each of the points. For example, in relation to anti-Muslim attitudes we have also reviewed:

Dunwoody PT, McFarland SG. Support for anti‐Muslim policies: The role of political traits and threat perception. Political psychology. 2018 Feb;39(1):89-106.

Uenal, F., Bergh, R., Sidanius, J., Zick, A., Kimel, S. and Kunst, J.R., 2021. The nature of Islamophobia: A test of a tripartite view in five countries. *Personality and Social Psychology Bulletin*, *47*(2), pp.275-292.

For Authoritarianism, we included a detailed discussion of:

Hanson K, O'Dwyer E, Lyons E. The national divide: A social representations approach to US political identity. European Journal of Social Psychology. 2021 Jun;51(4-5):833-46.
