# Supplementary material for: I won’t listen if I think we’re losing our way: How right-wing authoritarianism affects the response to different anti-prejudice messages
Source: PLoS One. 2023 Jan 17;18(1):e0280557. doi: 10.1371/journal.pone.0280557 (PMC9844905; doi:10.1371/journal.pone.0280557)
Supplement: Attachment — Submitted filename: rebuttal letter.docx [file pone.0280557.s002.docx]

We would like to thank all three reviewers for their feedback to adjust this manuscript. Below, we address all comments.

*Reviewer #2:*

*The article is well written and concise. Unfortunately, it adds little value to the published research.*

We appreciate these comments on the quality of the manuscript. We hope that our amendments add to the published research in a constructive way.

*Reviewer #3:*

*Title- I suggest correct the grammar ie I won’t listen*

We apologise for this; this was a direct quote from our pilot participants. We have amended the title.

*Americans may know what is meant by ‘the Muslim ban’ but I had to read all the way to p19 for an explanation of this. Please write for an international audience.*

Thank you for this comment. We have added a statement of clarification on both the abstract, and on line 61.

*The conclusion that RWA likely leads to resistance to all antiprejudice messages regardless of the source is overstated.*

We have removed the word “all” as there was a potential clarity issue here. What we meant was that the peer source likely doesn’t make much of a difference with people high in RWA.

*These findings can’t be generalised to e.g. messaging delivered by peers or influential figures.*

*Further, the experimental conditions are so far from the methods used by successful antiprejudice campaigns (such as Time to Change in England and Wales) I am not sure how useful the findings based on them are.*

We believe a bit of refinement is needed here. Here, we have added a bit of discussion on generalisation on lines 110, 112, 123,129, 383- pointing out two things: the social influence value, and the impact of anti-prejudice interventions against the topic:

-Social influence

From a social identity approach, the distinction between leader or peer based social influence is blurred. The *group* is the source of the influence, with prototypicality of a group member mattering more than anything else (Steffens et al, 2018). Therefore, how similar one is to the perceived group’s values, needs, desires, and state is where leaders and peers get their influence from, interacting with how people see the group. People are influential because they share group membership and embody what it means to be a group member. Our method of attempting to influence people via these prototypical Americans is against this approach. To clarify this, we have expanded a discussion on line **383 onward.**

Steffens, N. K., Haslam, S. A., Jetten, J., & Mols, F. (2018). Our followers are lions, theirs are sheep: How social identity shapes theories about followership and social influence. *Political Psychology*, *39*(1), 23-42.

-Different versions of prejudice

It is believed that prejudice toward things like mental health, disability, ageism, etc, likely function on a different level than things like immigrants, Muslims, Mexicans in the US, Indian/Pakistan views, Balkan prejudice etc. The former is more about symbolic threat- they’re a threat to how we live our lives, and the latter is a threat to our very existence (Rios et al, 2018). Therefore, we would have to adjust accordingly.

Anti-prejudice strategies, therefore, need to be tailored against what the threat is. This is why a contact intervention that seeks to build understanding between Muslims and Christians in Iraq fails to decrease prejudice between them outside of that specific context (Mousa et al, 2020), while a contact intervention that seeks to reduce ageism (Christian et al, 2014), or prejudice against people with mental health conditions likely would work (Morgan et al, 2018).

We’ve made some small adjustments on lines 110, 112 , 123 and 129 to address this to some extent. We have also mentioned this in limitations as well, line 451, 458.

Christian, J., Turner, R., Holt, N., Larkin, M., & Cotler, J. H. (2014). Does intergenerational contact reduce ageism: When and how contact interventions actually work?. *Journal of Arts and Humanities*, *3*(1), 1-15.

Morgan, A. J., Reavley, N. J., Ross, A., San Too, L., & Jorm, A. F. (2018). Interventions to reduce stigma towards people with severe mental illness: Systematic review and meta-analysis. *Journal of psychiatric research*, *103*, 120-133.

Rios, K., Sosa, N., & Osborn, H. (2018). An experimental approach to intergroup threat theory: Manipulations, moderators, and consequences of realistic vs. symbolic threat. *European Review of Social Psychology*, *29*(1), 212-255.

*Instead I think researchers should consider working with those who deliver such campaigns to evaluate them in a rigorous way; the delivery is much more sophisticated than what academics can deliver in an experiment, but academic evaluation is often lacking for social marketing campaign, so this would be a win win.*

We agree that would be ideal but here, we see this as more of a theoretical test. Does the contrast message shift people’s prejudice? Here, the answer is no, especially as those who likely needed the anti-prejudice most didn’t respond across all conditions. This is a consistent problem in anti-prejudice research, as highlighted in the paper.

On a more pessimistic view, the problem is very difficult to solve broadly. It is the lead author’s personal belief from working in government and in academia, and academic opinion, that most campaigns only shift people who have positive view to be more positive, while those who hold these views do not change. In order to address prejudice, schooling, shifts in society dynamics, and changes of *why* threats exist is far better. No message-based marketing campaign is likely to reduce prejudice in a meaningful way without additional work across more than one domain.

*Methods
I would not know what to make of a question about Americans and Muslims friendships- I would get stuck at the thought ‘what about all the Muslim Americans’? Were such questions subjected to cognitive testing first?*

The questions were largely taken from established scales, with very strong validity and reliability. While such a method might be a good idea for future research, it is largely out of scope for this proof of concept, as we were using an established scale previously used. This allows direct comparison with the other studies using this scale.

Pettigrew TF, Meertens RW. Subtle and blatant prejudice in Western Europe. European journal of social psychology. 1995;25(1):57-75.
